# Disentangling the Association Between Neurologic Deficits, Patient-Reported Impairments, and Quality of Life After Ischemic Stroke

**DOI:** 10.1212/WNL.0000000000206747

**Published:** 2023-03-28

**Authors:** Nadinda A.M. van der Ende, Sanne J. den Hartog, Joseph P. Broderick, Pooja Khatri, Johanna M.A. Visser-Meily, Nikki van Leeuwen, Hester F. Lingsma, Bob Roozenbeek, Diederik W.J. Dippel, and the IMS III Investigators

**Affiliations:** From the Departments of Neurology (N.A.M.v.d.E., S.J.d.H., B.R., D.W.J.D.), Radiology and Nuclear Medicine (N.A.M.v.d.E., S.J.d.H., B.R.) and Public Health (S.J.d.H., N.v.L., H.F.L.), Erasmus MC University Medical Center, Rotterdam, the Netherlands; Departments of Neurology and Rehabilitation Medicine (J.P.B., P.K.) and Emergency Medicine (J.P.B., P.K.), University of Cincinnati Gardner Neuroscience Institute, University of Cincinnati Academic Health Center, OH; and Center of Excellence for Rehabilitation Medicine (J.M.A.V.-M.) and Department of Rehabilitation, Physical Therapy Science & Sports (J.M.A.V.-M.), University Medical Center Utrecht, the Netherlands.

## Abstract

**Background and Objectives:**

The EuroQol Group 5-Dimension Self-Reported Questionnaire (EQ-5D) is a well-established instrument to assess quality of life and generates generic utility values for health states reported by patients, derived from assessments by the general public. We hypothesized that language problems and other nonmotor deficits are not captured as well as motor deficits by this system. We aimed to quantify the association between disabling neurologic deficits and the EQ-5D dimension scores and the utility score in patients with ischemic stroke.

**Methods:**

We used data of the Interventional Management of Stroke III trial. Missing data were imputed by multiple imputation. The association between neurologic deficits (individual NIH Stroke Scale [NIHSS] item scores) and the EQ-5D-3L (5 three-level dimension scores and utility score) at 90 days was assessed with ordinal logistic regression and Tobit regression, respectively. The explained variance of each model was estimated with Nagelkerke pseudo-R^2^ or R^2^.

**Results:**

In total, 525 surviving patients were included. Complete data on both the NIHSS and EQ-5D were available for 481/525 (91.6%) patients. At 90 days, 161/491 (32.8%) patients had aphasia and 226/491 (46.0%) patients had paresis of at least 1 limb. Limb paresis, facial palsy, sensory loss, and dysarthria explained most of the variance in all EQ-5D dimension scores and the utility score. In the utility score, 8.9% of the variance was explained by neglect, 10.0% by aphasia, 10.8% by hemianopia, and 17.5%–24.1% by limb paresis.

**Discussion:**

The impact of neurologic deficits on the EQ-5D in patients with ischemic stroke is mostly due to limb paresis, while the EQ-5D is less sensitive to other nonmotor deficits such as hemianopia, aphasia, and neglect. This may lead to overestimation of quality of life and, consequently, underestimation of the (cost-)effectiveness of treatments and interventions.

**Trial Registration Information:**

ClinicalTrials.gov. Unique identifier: NCT00359424.

Quality of life, as perceived by the lay public, based on patient-reported outcome measures is important for medical decision making and cost-effectiveness analyses.^1,2^ A frequently used patient-reported outcome measure for the assessment of quality of life is the EuroQol Group 5-Dimension Self-Reported Questionnaire (EQ-5D). The EQ-5D was designed as a generic instrument with dimensions relevant to all diseases and the general population. Its strength lies in the way its utility scores (i.e., weight assigned to health states, which represents the relative societal desirability of a particular health state) are derived from assessments by the general public, whose members are ultimately the persons to be involved in medical decision making, as a patient or a payer of tax and insurance premiums.

A generic instrument may not be able to capture all disabling aspects of a specific disease.^3-7^ This may not pose a problem, unless specific aspects are systematically underreported. It has been suggested that the EQ-5D may not cover the full range of deficits relevant to patients with ischemic stroke.^8^ Problems in motor functions due to limb paresis are obvious and, therefore, likely to be well reported on the EQ-5D by patients and by proxies. Other disabling neurologic deficits, such as hemianopia, sensory loss, aphasia, and neglect, may directly or indirectly lead to impairments and might not always be reported, especially because phrasing of the EQ-5D is generic and unsuited to focal neurologic deficits.

Neurologic deficits are often measured with the NIH Stroke Scale (NIHSS). Although the NIHSS and the EQ-5D are instruments with different purposes, one can imagine that disabling neurologic deficits should be reflected in the EQ-5D to a certain similar extent. The aim of this study was to quantify the association between disabling neurologic deficits measured with the NIHSS and the EQ-5D dimension scores and the utility score in patients with ischemic stroke.

## Methods

### Data

We used data from the Interventional Management of Stroke (IMS) III trial. This trial had data on the NIHSS and the EQ-5D at the same time point, 3 months after inclusion. The IMS III trial was a phase 3, multicenter, open-label clinical trial with blinded outcome assessment that evaluated the efficacy and safety of endovascular treatment plus intravenous thrombolysis compared with that of intravenous thrombolysis alone.^9,10^ Patients were enrolled from 58 international centers between August 2006 and April 2012, were aged 18–80 years, and had a moderate-to-severe ischemic stroke (NIHSS ≥ 10) before initiation of intravenous thrombolysis. For this study, we excluded patients who died before follow-up assessment at 90 days was performed. The study protocol and statistical analysis plan were previously published.^9,10^

### Standard Protocol Approvals, Registrations, and Patient Consents

The IMS III trial was approved by the ethics committee and research board of each participating center. Written informed consent was obtained from patients or their legal representative before enrollment in the trial. The IMS III trial was registered at clinicaltrials.gov (unique identifier: NCT00359424).

### NIHSS

The NIHSS is a 15-item neurologic examination scale, ranging from 0 to 42, with higher scores indicating more severe neurologic deficit.^11^ In the IMS III trial, the NIHSS was measured at 90 days after inclusion by study investigators who were not directly involved with acute treatment of the patient and who were blinded to treatment assignment.

### EQ-5D

In the IMS III trial, the EQ-5D with 3 levels was used. The EQ-5D consists of 5 dimensions: mobility, self-care, usual activities, pain/discomfort, and anxiety/depression.^2^ Each dimension has 3 levels coded from 1 to 3: 1, no problems; 2, some problems; and 3, extreme problems.^12,13^ On each dimension, patients chose the answer that best described their health status, which resulted in a 5-number sequence describing each level of each dimension. A sequence of 11111 represents perfect health and 33333 represents the worst possible health state. Therefore, the EQ-5D with 3 levels defines 243 (3^5^) possible health states. These health states were converted into a utility (i.e., weight assigned to a particular health state). These utility values are based on a country-specific value set, which is generated based on preferences given to EQ-5D health states by healthy inhabitants of a country.^12,13^ We used the utilities for the United States.^14^ Utilities range from 0 (death) to 1 (perfect health). Negative values can occur and are interpreted as situations worse than death.^15^

Assessment of the EQ-5D dimension scores was performed at 90 days after inclusion by study investigators who were not directly involved with acute treatment of the patient and who were blinded to treatment assignment. Information was obtained from the patient, if possible, and a proxy. For this study, we used the EQ-5D dimension scores as indicated by the patient. If not available, we used the EQ-5D dimension scores as indicated by their proxy. For some patients, EQ-5D dimension scores for both an in-person and telephone assessment were available. In those cases, the scores from the in-person assessment were used.

### Statistical Analysis

Clinical characteristics of patients according to availability of the NIHSS and EQ-5D at 90 days were compared using descriptive statistics. We assessed the association between neurologic deficits (NIHSS sum score and individual NIHSS item scores) and each EQ-5D dimension score with univariable ordinal logistic regression. For the EQ-5D utility score, censoring from above takes place, which means cases with a value at or above some threshold (i.e., utility of 1), all take on the value of that threshold, so that true value might be equal to the threshold, but it might also be higher.^16,17^ This will skew the distribution, and therefore, the association between the NIHSS (i.e., sum score and individual item scores) and the EQ-5D utility score was assessed with Tobit regression models.^16,17^ After constructing the regression models as appropriate, Nagelkerke pseudo-R2 for the EQ-5D dimension scores (ordinal outcome) and R2 for the EQ-5D utility score (continuous outcome) were estimated to quantify the explained variance in outcome by the NIHSS (i.e., sum score and individual item scores). Because proxies tend to score patients as more severely impaired,^18-20^ data were also analyzed stratified by EQ-5D assessments completed by patients and EQ-5D assessments completed by proxies in a sensitivity analysis.

Missing data were imputed by multiple imputation by chained equations based on relevant covariates including outcomes.^21^ Statistical analyses were performed with R statistical software (version 4.0.5).

### Data Availability

Anonymized trial data and methods that support our finding are available upon request (ninds.nih.gov/Current-Research/Research-Funded-NINDS/Clinical-Research/Archived-Clinical-Research-Datasets).

## Results

**Figure 1:**
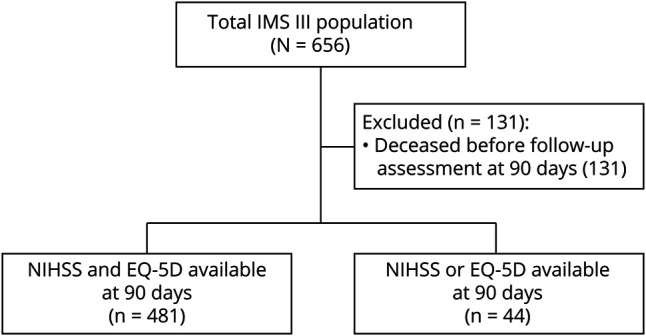
Flowchart of IMS III Patients Selected for Analysis EQ-5D = EuroQol Group 5-Dimension Self-Report Questionnaire; IMS = Interventional Management of Stroke; NIHSS = NIH Stroke Scale.

Among the 525 surviving patients included in this study, 481/525 (91.6%) patients had complete data on both the NIHSS and EQ-5D (Figure 1). Characteristics of patients at baseline and outcomes at 90 days are presented for patients with data on both the NIHSS and EQ-5D and for patients with a missing NIHSS and/or EQ-5D (Table and eTable 1, links.lww.com/WNL/C559). Baseline characteristics of the 2 groups were similar. The median modified Rankin scale score at 90 days was 2 (interquartile range [IQR] 1–3) for patients without missing values for both the NIHSS and EQ-5D at 90 days. The median modified Rankin scale score at 90 days was 4 (IQR 1–4) for patients with a missing value on the NIHSS and/or EQ-5D at 90 days. At 90 days, 161/491 (32.8%) patients had aphasia and 226/491 (46.0%) had paresis of at least 1 limb (eTable 1). Of the 161 patients with aphasia, 62 patients (38.5%) had aphasia without any limb paresis.

**Table:**
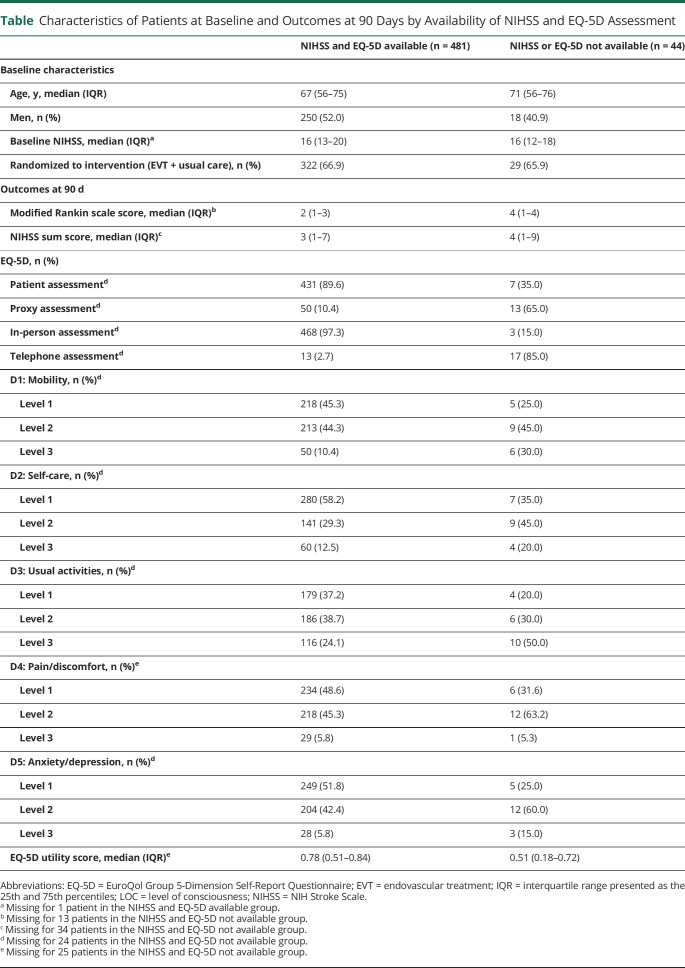
Characteristics of Patients at Baseline and Outcomes at 90 Days by Availability of NIHSS and EQ-5D Assessment

**Figure 2:**
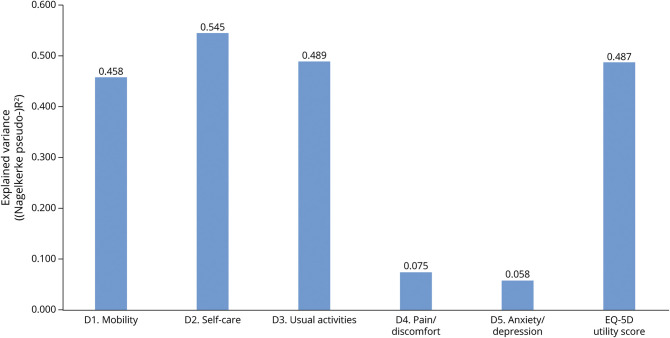
Explained Variance of the NIHSS Sum Score on the EQ-5D Dimension Scores and on the Utility Score EQ-5D = EuroQol Group 5-Dimension Self-Report Questionnaire; NIHSS = NIH Stroke Scale.

Patients with higher NIHSS scores reported more problems on each EQ-5D dimension score (eFigure 1, links.lww.com/WNL/C559). The NIHSS sum score explained 45.8% of the variation in mobility, 54.5% in self-care, 48.9% in usual activities, 7.5% in pain/discomfort, 5.8% in anxiety/depression, and 48.7% in utility score (Figure 2).

**Figure 3:**
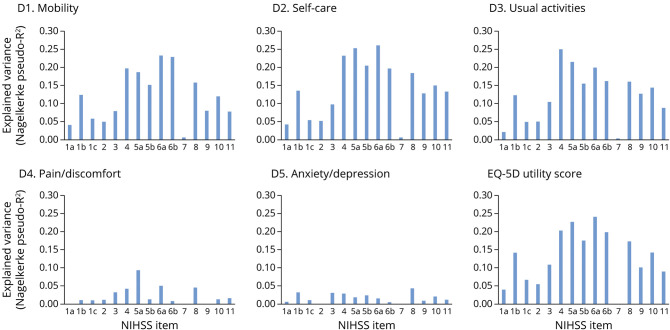
Explained Variance of NIHSS Items on the EQ-5D Dimension Scores and on the Utility Score EQ-5D = EuroQol Group 5-Dimension Self-Report Questionnaire; NIHSS = NIH Stroke Scale; 1a, level of consciousness (LOC); 1b, LOC questions; 1c, LOC commands; 2, best gaze; 3, visual; 4, facial palsy; 5, motor arm left (a) and right (b); 6, motor leg left (a) and right (b); 7, limb ataxia; 8, sensory; 9, best language; 10, dysarthria; and 11, extinction and inattention.

Limb paresis, facial palsy, sensory loss, and dysarthria explained most of the variance in all EQ-5D dimension scores and the utility score (Figure 3). In pain/discomfort, aphasia explained 0% of the variance and limb paresis explained 0.8% (item 6b) to 9.3% (item 5a) of the variance. In anxiety/depression, aphasia explained 0.9% of the variance and limb paresis explained 0.5% (item 6b) to 2.4% (item 5b) of the variance. Hemianopia explained 8.0% of the variance in mobility, 9.8% in self-care, 10.6% in usual activities, 3.3% in pain/discomfort, and 3.2% in anxiety/depression. Neglect explained 7.9% of the variance in mobility, 13.4% in self-care, 8.9% in usual activities, 1.7% in pain/discomfort, and 1.3% in anxiety/depression. The explained variance in the utility score was 8.9% for neglect, 10.0% for aphasia, 10.8% for hemianopia, and 17.5% (item 5b) to 24.1% (item 6a) for limb paresis.

In the sensitivity analysis, 441 EQ-5D assessments were completed by patients and 386 EQ-5D assessments were completed by proxies (eFigure 2, links.lww.com/WNL/C559). Data were analyzed stratified by EQ-5D patient assessment and EQ-5D proxy assessment, which showed similar results as the main analysis (eTable 2, eFigures 3 and 4).

## Discussion

We quantified the association between disabling neurologic deficits measured with the NIHSS and the 5 EQ-5D dimension scores and the EQ-5D utility score in patients who had an ischemic stroke. We hypothesized that language problems and other nonmotor deficits are not captured as well as motor deficits by this system. This study showed that the explained variance of neurologic deficits was lower on the dimensions pain/discomfort and anxiety/depression than on the other EQ-5D dimension scores and the utility score. Motor deficits caused by limb paresis explained more of the variance on the EQ-5D dimension scores and the utility score than other nonmotor deficits such as hemianopia, aphasia, and neglect.

Our results are in line with previous research showing that the EQ-5D dimensions mobility, self-care, and usual activities were more strongly correlated with the modified Rankin scale and Barthel index than the EQ-5D dimensions pain/discomfort and anxiety/depression in patients with ischemic stroke.^22^ Another study showed that the effect of endovascular treatment on the EQ-5D utility score was relatively small compared with other clinical outcomes, and they did not find a treatment effect on the EQ-5D dimensions pain/discomfort and anxiety/depression.^8^

We used the NIHSS to quantify the association between neurologic deficits and the EQ-5D dimension scores and the utility score. Although the NIHSS and the EQ-5D are instruments with different purposes, one can imagine that disabling neurologic deficits should be reflected in the EQ-5D to a certain extent. We expected that the dimensions pain/discomfort and anxiety/depression would be influenced by aphasia because we assumed aphasia may limit participation, might cause anxiety, and might cause cognitive and emotional discomfort.^23^ An explanation for the limited influence of aphasia on the EQ-5D could be that quality of life of patients is not influenced by aphasia. However, more likely is that the EQ-5D is less sensitive for aphasia because limitations of the EQ-5D regarding the reflection of impact on health status have been demonstrated for other diseases, such as psoriasis, macular degeneration, and psychological disorders.^3-5^ Another problem is patients who are not aware of their deficits, which occurs with neglect, hemianopia, and some forms of aphasia. Because they are unaware of their deficits, they will not report it. Hemianopia and neglect explained only 9%–10% of the variance in the utility score, although these symptoms may severely affect quality of life. Of importance, because neglect represents only a maximum of 2 points on the NIHSS, the impact of neglect could be underestimated in this study. Underreporting of symptoms and discomfort on the EQ-5D dimension scores may lead to an overestimation of the utility score. For example, if patients report no problems on all dimensions (“11111”), but have some discomfort (“11121”), their utility score should be 0.827 instead of 1.^14^ This can have important consequences because an overestimation in utility score can result in an underestimation of the (cost-)effectiveness of stroke treatments compared with treatments of other diseases. Future research should assess whether this causes an overestimation of quality of life. First, research should compare the sensitivity of the EQ-5D with disease-specific patient-reported instruments to confirm our findings. Next, it should be evaluated whether adding a modified instruction to interpret discomfort in a different, more broader sense to incorporate problems such as not being able to see, speak, or understand language well, or to be aware of one's own body, and the anxiety that may accompany these deficits would improve the assessment of quality of life through the EQ-5D.

Quality of life can be measured with generic measures or disease-specific measures, such as the Stroke-Specific Quality of Life, Stroke Impact Scale or STATIS-Stroke for ischemic stroke.^24^ Stroke-specific quality-of-life measures could be more suitable as outcome measures in stroke trials than generic quality-of-life measures because they have higher correlations with commonly used stroke outcome measures (i.e., Barthel index, modified Rankin scale, and NIHSS).^25^ However, because stroke-specific quality-of-life measures cannot be used to achieve direct comparisons across diseases and therapies, generic quality-of-life measures as the EQ-5D are essential.

This study has some limitations. The study population consisted mainly of patients with anterior circulation ischemic stroke. Therefore, only a few patients had ataxia, and we cannot draw any conclusions about the association between ataxia and the EQ-5D. We expect that ataxia does substantially influence the EQ-5D, especially on the dimensions mobility, self-care, and usual-activities, but this should be confirmed in future research. In our study, patients with missing values on the NIHSS and/or EQ-5D at 90 days had worse outcomes compared with patients without missing values. It is worrisome that missing values on the NIHSS and/or EQ-5D might be associated with outcomes. However, because we used multiple imputation based on relevant covariates including outcomes, which provides less biased estimates compared with excluding those patients, we expect that those missing values did not influence our results.^26^ Another limitation is that the EQ-5D with 3 levels was used instead of the newer 5-level version. However, we do not expect a different conclusion because phrasing of both versions is similar. Finally, because the median NIHSS was 3 (IQR 1–7) at 90 days, some patients had more than 1 type of neurologic deficit, which might have influenced our results. For example, patients with aphasia can also have motor weakness, which might be the reason for the association and explained variance of aphasia on mobility. However, this suggests that the impact of aphasia on the EQ-5D might be lower than we found. Moreover, 62/161 (38.5%) patients with aphasia had aphasia without any limb paresis.

To conclude, the impact of neurologic deficits on the EQ-5D in patients with ischemic stroke is mostly due to limb paresis, while the EQ-5D is less sensitive to other nonmotor deficits such as hemianopia, aphasia, and neglect. This may lead to overestimation of quality of life and, consequently, underestimation of the (cost-)effectiveness of treatments and interventions.

## Glossary

EQ-5D: EuroQol Group 5-Dimension Self-Reported Questionnaire
IMS: Interventional Management of Stroke
IQR: interquartile range
NIHSS: NIH Stroke Scale
